# Enlightening Hidden Nursing Care in Nurse-Led Clinics and See & Treat: An Observational Multicenter Protocol Study in Italy

**DOI:** 10.3390/nursrep14040274

**Published:** 2024-11-29

**Authors:** Francesco Zaghini, Valeria Caponnetto, Manuele Cesare, Marco Di Nitto, Ilaria Marcomini, Paolo Iovino, Yari Longobucco, Annamaria Bagnasco, Loreto Lancia, Duilio Fiorenzo Manara, Laura Rasero, Gennaro Rocco, Giancarlo Cicolini, Beatrice Mazzoleni, Maurizio Zega, Walter Sermeus, Jonathan Drennan, John Welton, Loredana Sasso, Rosaria Alvaro

**Affiliations:** 1 Department of Biomedicine and Prevention, University of Rome Tor Vergata, 00133 Rome, Italy; francesco.zaghini@uniroma2.eu (F.Z.); rosaria.alvaro@uniroma2.it (R.A.); 2 Department of Life, Health and Environmental Sciences, University of L’Aquila, 67010 Coppito, Italy; valeria.caponnetto@univaq.it (V.C.); loreto.lancia@univaq.it (L.L.); 3 A. Gemelli IRCCS University Hospital Foundation, 00168 Rome, Italy; 4 Department of Health Sciences, University of Genoa, 16132 Genoa, Italy; marco.dinitto@unige.it (M.D.N.); annamaria.bagnasco@unige.it (A.B.); l.sasso@unige.it (L.S.); 5 Faculty of Medicine and Surgery, Vita-Salute San Raffaele University, 20132 Milan, Italy; marcomini.ilaria@unisr.it (I.M.); manara.duilio@unisr.it (D.F.M.); 6 Department of Health Sciences, University of Florence, 50143 Florence, Italy; paolo.iovino@unifi.it (P.I.); yari.longobucco@unifi.it (Y.L.); l.rasero@unifi.it (L.R.); 7 International Center for Nursing Research Montianum (CIRIM), Department of Biomedical Sciences, Catholic University Our Lady of Good Counsel, 1000 Tirana, Albania; g.rocco@unizkm.al; 8 Center of Excellence for Nursing Scholarship (CECRI), Board of Nursing (OPI) of Rome, 00136 Rome, Italy; 9 Department of Innovative Technologies in Medicine & Dentistry, G. d’Annunzio University of Chieti—Pescara, 66100 Chieti, Italy; g.cicolini@unich.it; 10 Department of Biomedical Sciences, Humanitas University, 20090 Milan, Italy; beatrice.mazzoleni@hunimed.eu; 11 Isola Tiberina Hospital—Gemelli Isola, A. Gemelli IRCCS University Hospital Foundation, 00168 Rome, Italy; maurizio.zega@fbf-isola.it; 12 Leuven Institute for Healthcare Policy, KU Leuven, 3000 Leuven, Belgium; walter.sermeus@kuleuven.be; 13 UCD School of Nursing, Midwifery and Health Systems, University College Dublin, Belfield, D04 V1W8 Dublin 4, Ireland; jonathan.drennan@ucd.ie; 14 Division of Health Systems, Leadership, and Informatics, University of Colorado College of Nursing, Aurora, 80045 CO, USA; john.welton@cuanschutz.edu

**Keywords:** health care costs, health policy, hidden nursing care, nurse-led clinics, protocol studies, reimbursement, See & Treat

## Abstract

**Background/Objectives:** The limited and inconsistent adoption and regulation of nurse-led clinics (NLCs) and “See & Treat” (S&T) services in Italy needs to be explored considering their value towards patients’ outcomes acknowledged in the literature. This study aims to explore the phenomenon of hidden nursing activities (HNAs) in these settings, hypothesizing that features and activities performed in these settings are heterogeneous across the country and widely underreported or attributed to other professionals than nurses. HNAs are hypothesized to be associated with a poor work environment climate and nurses’ low job satisfaction. **Methods:** A multicenter, cross-sectional study will be conducted across exclusively nurse-led NLC and S&T services in public health care facilities in Italy. Data collection will involve inputs from organization or nursing managers, coordinators, head nurses, and employed nurses. Information will be gathered on organizational structure, service provision, access modalities, nurses’ perceptions of their work environment, and the health care activities performed. Surveys will be distributed online to collect retrospective data in 2023 and via paper to collect 1-month prospective data about services’ activities. **Expected results:** This study is expected to reveal HNAs in NLC and S&T, with implications for policy, resource allocation, reimbursement models, and patient outcomes, ultimately supporting healthcare reforms and enhancing nursing’s visibility and impact in Italy. The findings will be essential for guiding health care resource allocation and shaping educational and regulatory policies that recognize and formalize the role of nurses in advanced practice. Policymakers could leverage the findings of this study to promote the development of standardized taxonomies, making nursing contributions more visible and measurable. Ultimately, this research will highlight the value of nursing care in NLC and S&T settings, providing an evidence base to drive policy changes that improve both health care outcomes and resource efficiency. **Conclusions:** This study lays the groundwork for health care policy reforms by advocating for the recognition, measurement, and funding of nursing contributions, ultimately enhancing patient outcomes and the sustainability of health systems.

## 1. Introduction

Recognizing the critical roles nurses play and the tangible impact of nursing care on patient outcomes is essential for driving meaningful changes in both policy development and practice. Such recognition not only provides society with a clearer and more accurate perspective of the nursing profession but also enables a more responsive health care system that can swiftly adapt to citizens’ evolving health care needs [1,2]. In an era when the sustainability of health systems is under intense scrutiny, revealing the hidden dimensions of the nursing profession can clarify health care dynamics, making them more understandable and transparent. This can, in turn, facilitate the adoption of policies and practices that ensure health systems are both effective and efficient. However, a persistent lack of understanding of nursing care and activities, along with the failure to adopt standardized nursing terminology in various care settings, complicates efforts to demonstrate the true impact of nursing care on patient outcomes across diverse environments [3]. This lack of visibility leads to the phenomenon of hidden nursing care, where nursing activities are either unrecognized, inadequately documented, or misattributed within health care systems. These hidden tasks, though critical to patient outcomes, are undervalued or even credited to other health care roles, diminishing the visibility and proper compensation for nursing contributions. The consequences of this phenomenon are far-reaching, including organizational ambiguity, the inappropriate assignment of nursing tasks, and the misattribution of payments for these services to other health care professionals [4]. For example, wound care provided by nurses may be registered and reimbursed as if performed by physicians, distorting the true nature of care delivery and misallocating resources. This misrepresentation creates problems for patients, such as longer waiting times for appointments, and undermines public health efforts by obscuring the vital contributions of nursing to health care systems. Addressing hidden nursing care is therefore a policy imperative for ensuring that nursing contributions are fully recognized and appropriately integrated into health care systems. By making the impact of nursing activities visible and accountable, policymakers and health care administrators can make informed decisions that enhance the efficiency and effectiveness of health care delivery.

## 2. Background

Epidemiological changes in recent decades, particularly the rise in non-communicable chronic diseases and population aging, have significantly increased the demand for complex care, prompting a global paradigm shift in care provision [5]. At the same time, advances in biomedical science have underscored the need for highly skilled nurses and specialized areas of expertise to ensure the delivery of high-quality care [6,7,8]. This shift has led to significant intellectual and professional development, especially in English-speaking countries, resulting in the diversification of nursing post-graduate curricula and the emergence of advanced practice nurses, clinical nurse specialists, and clinical nurse leaders [9]. However, these roles have not been consistently acknowledged in all countries, and their responsibilities often remain unclear [10,11].

The increased capabilities of professionals trained through postgraduate programs have been well documented, with clear evidence of favorable outcomes for population health [12]. To support these evolving roles, new organizational models, such as nurse-led clinics (NLCs) and emergency department See & Treat (S&T) models have emerged [13,14], allowing specialist nurses to serve communities more efficiently in both hospital and primary care settings [15,16]. Research confirms that NLCs, operating across various specialties, yield outcomes equal to or better than standard care, particularly in areas such as mortality, medication adherence, disease and symptom management, quality of life, and patient satisfaction [15]. Likewise, the S&T model has demonstrated effectiveness in reducing emergency department overcrowding, patient wait times, lengths of hospital stay, and costs, while improving patient safety and satisfaction [13,17,18]. Like for educational pathways, despite these positive outcomes, the integration of such models into health care systems has varied significantly across countries. International guidelines for advanced nursing practice exist, but postgraduate training programs remain inconsistent [19].

In Italy, postgraduate degrees such as the Master of Science in Nursing (MSN) are required for nurse leadership positions, while 1-year specialization courses offer the opportunity to develop advanced clinical competencies. However, the recognition of these qualifications is inconsistent, with roles often poorly regulated and lacking formal contractual or economic acknowledgment [20]. While clinical specialization certifications are not mandatory for employment in specific settings, acquiring them does not always guarantee a corresponding job position or salary upgrade, even for nurses working in their area of specialization. Furthermore, organizational models like NLCs and S&T are not uniformly regulated by national law in Italy, often being implemented through regional or local initiatives. This fragmentation results in inconsistencies across regions and health care facilities. Although the S&T model has gained more traction, issues persist regarding the standardization of services and the absence of a unified taxonomy for nursing activities. This has led to the erroneous attribution of nursing tasks to other health care professionals, a problem that has been documented for decades in hospital settings [21], perpetuating the phenomenon of hidden nursing activities (HNAs). HNAs not only conceal the value that nurses contribute to public health but also lead to frustration among nurses [10,11], adversely impacting their well-being, job satisfaction, and ultimately the health of the populations they serve [22,23].

Accurately capturing and recognizing the contributions of nursing is crucial for advancing health care management and informing policy reform. HNAs, which are often overlooked in health care systems, need to be thoroughly examined to ensure the full scope of nursing’s role is visible and recognized. Moreover, the financial cost of nursing care is frequently bundled into the fixed component of overall health care expenditures, including NLC and S&T services. However, little is currently known about the true intensity and actual cost of direct nursing care [7,24]. To bridge this gap, a comprehensive analysis of nursing activities in NLC and S&T settings is essential.

### 2.1. Aims

This study aims to investigate the phenomenon of HNAs in Italian NLCs and S&T settings by examining HNAs’ context, features and amount, associated costs, misattribution to other professionals, and associated nurses’ perception of the work environment and job satisfaction.

The principal objectives of the study are to:

(a) identify and map the NLCs and S&T services run by nurses in Italy, documenting their organizational characteristics and describe the sociodemographic (e.g., age, gender) and work characteristics (e.g., years of experience, employment contract type) of nurses working in NLCs and S&T services, by the end of the study period; (b) describe and categorize the patient admission modalities for NLCs and S&T services, quantifying each modality used over a 30-day observation period; (c) map and quantify the specific nursing activities performed within these settings; (d) determine the presence and use of unique codes for reporting nursing activities, assessing their availability and application in NLCs and S&T services; and (e) verify and document the cost centers and reimbursement methods associated with NLCs and S&T activities, establishing a comprehensive report on financial allocation and reimbursement practices.

The secondary objectives are to:

(a) quantify and analyze untraceable nursing activities (e.g., health education, health promotion, tertiary prevention etc.) performed by nurses within NLCs and S&T settings, specifying the volume and types of unrecorded activities over the study period; (b) calculate the nursing care cost (in man-hours) associated with untraceable services provided to patients or caregivers, establishing a baseline estimate for these activities; (c) examine the work environment, conditions, and job satisfaction levels of nurses within NLCs and S&T services, using validated scales to measure these factors within the study timeframe.

### 2.2. Study Hypotheses

**H1:**  *In Italian NLCs and S&T services, organizational characteristics, activities performed, and related reporting codes and cost centers are widely heterogeneous across facilities.*

**H2:**  *Nurses in NLCs and S&T settings spend considerable time on unreported activities, such as health education, which are not adequately documented or reimbursed, impacting patient care and resource allocation.*

**H3:**  *In Italian NLCs and S&T services, most nursing activities and related costs are attributed to other professionals.*

**H4:**  *HNAs are linked to poor work environment climate, lower job satisfaction, and increased frustration among nurses.*

## 3. Materials and Methods

### 3.1. Design and Study Approach

This protocol is designed as a multicenter, retrospective cross-sectional, and prospective observational study. The observational approach allows for the collection of real-world data on nursing practices within NLCs and S&T services. By combining retrospective, cross-sectional, and prospective observational data, the study aims to capture comprehensive insights into the workflows, service characteristics, and unrecognized nursing activities within these settings, without any intervention. This approach enables the research team to analyze existing practices across diverse health care facilities.

### 3.2. Study Setting, Sampling, Inclusion, and Exclusion Criteria

The study will be conducted in Italian NLCs, operating in both community and hospital settings. These include hospitals, university hospitals, local health facilities, and local health care area facilities (locally referred to as AUSL/ASST), which provide exclusively nurse-led services, as well as hospital and/or community settings where S&T services are available, such as emergency rooms and community homes.

All public nurse-led facilities and S&T services across the national territory that are willing to participate will be included in the study. Private NLCs and S&T services will be excluded, even if they are affiliated or accredited by the National Health System. Given the absence of national data on these services, a sample size estimate is not possible. Therefore, the study will gather all available data on service flows from the observation period (year 2023) and prospective observational data over a 30-day period.

A letter of intent will be sent to all facilities that meet the inclusion and exclusion criteria, inviting them to participate in the study. Facilities that agree to participate will be contacted and provided with detailed instructions on how to engage in the research activities.

### 3.3. Variables

The variables will be collected on three levels: nursing managers, head nurses, and nurses. The following data will be investigated: number of nurse-led facilities and S&T services present in Italy, number and sociodemographic characteristics of nurses employed in these services, organizational characteristics (e.g., daily availability, details on educational background and work experience of nurses), procedures for accessing these services, number and types of nursing activities provided, activities performed on patients and related code attribution (if available), daily time dedicated to these activities, methods for documenting nursing activities, methods for recording prescribed activities (e.g., demand, number of cycles, total number of activities, etc.), total cost of interventions attributed to the NLCs services/S&T activities, work environment climate and work conditions perceived by nurses, and job satisfaction levels of nurses.

### 3.4. Identification of Facilitators

Facilitators will be identified in collaboration with the nursing managers of the participating health care facilities. These facilitators will undergo a specific training session designed to ensure they effectively manage the study and strictly adhere to its protocols. Their key responsibilities will include informing the nurses working in the NLCs and S&T services about the study’s nature and objectives. Additionally, they will coordinate and support the data collection process, ensuring smooth operations, and addressing any local issues that may arise during the course of the research. This phase of the study will last 6 months, from June to November 2024, due to potential enrollment difficulties.

### 3.5. Data Collection

Data collection will be conducted through surveys at three levels: (1) company management or nursing management, (2) head nurses/nurses in charge of the NLCs or S&T services, (3) and nurses working in the NLCs/S&T services during the study period.

Moreover, enrolled nurses will be provided with paper forms specifically developed to collect the activities performed in the NLCs or S&T settings.

In particular, for the facilities enrolled in the study, the managing director or the nursing director will be asked to complete an online survey providing information on the characteristics of the health care facility during the study period (e.g., number of services, staffing levels), as well as data regarding health care activities provided to patients in NLC/S&T services (retrospective data from 2023). Data collection will occur retrospectively through the retrieval of administrative information from the services by consulting existing datasets in management control services.

Coordinators/nurses in charge of NLCs services will also be asked to complete an online survey detailing the health care services provided to patients during 2023.

Nurses will be asked to complete two tools. The first is an online questionnaire to collect cross-section data regarding their sociodemographic data, work conditions, and the Practice Environment Scale of the Nursing Work Index (PES-NWI) to evaluate the work environment [25]. In particular, the PES-NWI consists of nine items through which participants can indicate their level of agreement with each statement using a four-point Likert scale, ranging from 1 = “strong disagreement” to 4 = “strong agreement”, and in the validation study, the reliability of each component ranged from a minimum Cronbach’s α of 0.80 to a maximum value of 0.92. The second instrument of data collection is a prospective sheet documenting nursing services performed over a 30-day period. Prospective data collected in paper will be shared with authors in pdf format by the coordinators of services. Data will be inputted in Microsoft Excel^®^ to be analyzed. The list of activities included in the data collection sheet was developed by the research group after reviewing the literature to identify the most frequent activities in these care settings. This sheet includes a predefined list of activities and allows nurses to add other activities using free text.

All data collection links and tools will be distributed by the nursing managers of each facility to designated facilitators, who will then share them with the relevant participants.

This phase of the study will last 6 months, from July to December 2024.

**Figure 1:**
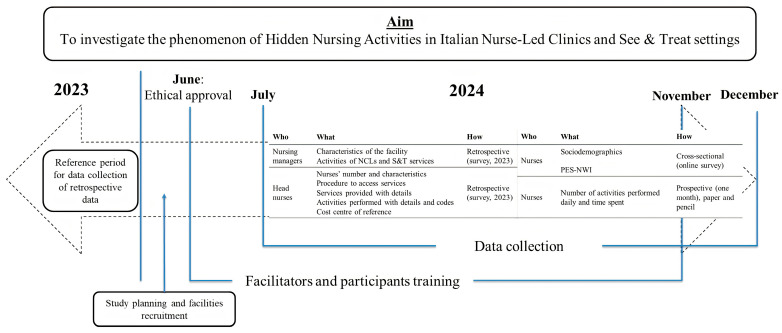
Study aim and overall methodological features.

An overview of the study’s aim and the overall methodological features is outlined in Figure 1.

### 3.6. Data Analysis

To address the study’s objectives, descriptive statistics will be applied to data collected from all surveys. These statistics will include frequency, percentage, mean, and standard deviation. Data will be analyzed in clusters based on region, diagnosis/procedure, and volume, allowing for a more in-depth understanding of the workflow and the ability to quantify the cost of individual activities.

To assess heterogeneity across facilities, organizational characteristics, activities performed, and related reporting codes and cost centers will be described for each enrolled service and differences will be explored using inferential statistics, as appropriate. Activities described by nurses in free-text responses will undergo qualitative evaluation, with the authors grouping them into relevant categories and then analyzing them using frequencies.

Underreporting of nursing activities will be explored by describing the amount and type of nursing activities documented in the prospective period of observation and the profession or cost center to which these were attributed.

For cost estimation, the average time dedicated to nursing interventions (in man-hours) will be multiplied by the number of services provided during the observation period. This will include average hourly compensation for the category of service and average cost per service. The resulting cost estimate will then be compared with the total reimbursement received from health care centers for each service evaluated.

To verify the association between HNAs and work environment climate and job satisfaction, multiple linear regressions will be performed.

Missing data will be limited through the role of facilitators. Data patterns will be analyzed to guide the selection of appropriate imputation methods (such as mean, median, multiple, or mode imputation) based on variable type and distribution. Inconsistent entries will be identified and corrected through cross-validation checks. Additionally, sensitivity analyses will be performed to verify the robustness of the results. All analyses will be conducted using the SPSS Version 25^®^ statistical package.

### 3.7. Presentation of Results

The study’s findings will be presented through a series of tables and graphs to facilitate a clear and comprehensive understanding of the data. Tables will be used to summarize descriptive statistics, such as frequencies, means, and cost estimates of nursing activities. Graphs—including bar charts, and line graphs—will illustrate comparative data across facilities and regions. Flow diagrams will also be used to map patient care pathways and nursing workflows within NLC and S&T services. These visual aids are intended to enhance the accessibility and interpretability of the results for both health care practitioners and policymakers.

### 3.8. Ethical Considerations, Acquisition of Informed Consent, and Data Processing

The protocol for this study was approved by Lazio Regional Ethics Committee 2 (Protocol 21.24 CET2 utv_ptv) on 20 June 2024. Any future amendments to the protocol will be promptly submitted to the same ethics committee for approval. Local ethical approvals will also be sought for participating centers if necessary. The study will be conducted in full compliance with international (EU Directive 2001/20/EC) and national legislation (DM 15 July 1997; Legislative Decree 211/2003; Legislative Decree 200/2007) regarding clinical trials, and it will adhere to the principles of the Declaration of Helsinki and Good Clinical Practice (GCP) guidelines.

Upon receiving the link to the online surveys, participants will be able to access an information sheet that provides details on the study’s purpose, methods, expected benefits, and potential risks. The information sheet will also clarify that non-participation or withdrawal will not result in any prejudice or harm to the participants. Only after ticking the box to provide informed consent to the study and personal data processing will participants be allowed to continue completing the questionnaire.

Data from the surveys will be collected via LimeSurvey^©^ version 6.0.0, and access credentials to the platform will be limited to the researchers directly involved in the study. To ensure participant privacy, data will be pseudonymized by assigning each participating center an alphanumeric code, which will be randomly generated by the research team using Microsoft Excel^®^ and applied across all data collection forms.

## 4. Expected Results

This study protocol outlines a comprehensive approach to uncovering and documenting HNAs within NLCs and S&T services across Italy. A wide heterogeneity across facilities is expected regarding features, activities performed and nurses’ characteristics and education [10,11,26]. By examining a range of nursing activities that are often unrecognized or misattributed, this study aims to provide a detailed view of nursing contributions within these specialized care settings. Through the collection of retrospective data from 2023 and prospective data over a 30-day period, this study is expected to yield detailed information on the types and frequency of nursing activities performed in NLCs and S&T services. The study aims to capture how nursing tasks contribute to patient care, including direct care, health education, preventive care, and administrative functions. These data will provide a clear picture of the scope of nursing responsibilities and identify any areas where nursing activities are unrecorded or misclassified. Details on costs of these activities are also expected, considering the limited availability of data in the literature on this topic [4].

Finally, the exploration of work environment climate and nurses’ job satisfaction is expected to confirm association hypotheses between these variables and HNAs [22,23].

## 5. Discussion

This study is expected to provide essential insights into HNAs within NLCs and S&T services, with substantial policy, strategic, economic, clinical, and social implications. Politically, the results will support the development of national policies and regulatory frameworks that formally recognize and fund advanced nursing roles, thereby enhancing the visibility and accountability of nursing contributions within the health care system [26]. Strategically, the study is anticipated to reveal specific domains where nursing practices significantly enhance care delivery efficiency, which could guide resource allocation, workforce planning, and the integration of advanced nursing competencies within health care teams at both organizational and governmental levels [7]. Economically, the quantification of nursing contributions could inform adjustments to reimbursement models, ensuring that nursing activities are accurately attributed and compensated [4]. Such adjustments would promote optimal financial stewardship within health care systems, allowing for a more efficient allocation of public health funds and contributing to the sustainability of health services. Clinically, by documenting and analyzing the scope of nursing activities, this study aims to highlight the critical role of nursing in improving patient outcomes, reducing emergency department congestion, and enhancing patient satisfaction [6,7,8,12]. Socially, this study aims to elevate the professional status and job satisfaction of nurses by recognizing and validating their contributions, which can lead to improved morale, retention, and a stronger nursing workforce. Additionally, by improving health care efficiency and patient care quality, the findings may indirectly foster public trust and confidence in the health care system. These findings could serve as a foundational evidence base for health care reforms aimed at better integrating nursing activities into financial, operational, and policy frameworks, thereby supporting a more sustainable and effective health care system in Italy.

The multicenter observational design of this study, which includes both retrospective and prospective data collection, is a key strength, enabling a robust analysis of nursing practices across diverse health care settings. By focusing on both community and hospital-based NLCs and S&T services, the study is well-positioned to capture variations in nursing practices and workflows. Additionally, the use of standardized tools, such as the PES-NWI, will allow for consistent and reliable data on the work environment and job satisfaction among nurses, contributing to a deeper understanding of the nursing context in Italy.

### 5.1. Limitations of the Study

As with any observational study, there are limitations that may impact the generalizability of the findings. First, the reliance on self-reported data could introduce response biases or inaccuracies, particularly in the retrospective data collection phase. Self-reported surveys are subject to several limitations, including potential biases such as recall inaccuracies and social desirability effects [27]. To mitigate these biases, we will incorporate cross-verification with available administrative records where possible, which will allow us to validate key data points and reduce recall and social desirability effects. Moreover, the heterogeneity in both the services under evaluation and the characteristics of the respondents can introduce additional complexity. Variations in service provision and individual differences among participants can lead to inconsistencies in the reported data, complicating the generalizability and interpretability of the findings. To address this, we will conduct subgroup analyses by service type and respondent profile, identifying and accounting for differences in service provision and participant characteristics. This approach will enhance the interpretability of our results across diverse service contexts. Additionally, variability in documentation practices across regions and facilities may create inconsistencies in data quality. To improve data collection, we will provide comprehensive training sessions for facilitators and participants on documentation procedures, including standardized data entry templates, and implement routine data quality checks to promptly address discrepancies.

These factors will be carefully considered to accurately assess and interpret survey results. The research group will describe the organizational characteristics of the services involved and the professional attributes of the respondents in detail, ensuring transparency and reproducibility of the results.

### 5.2. Future Research Directions

The findings from this study will provide a foundation for future research that could explore the impact of HNAs on patient outcomes and health care efficiency. Subsequent studies could examine specific strategies to standardize nursing documentation, facilitating a more accurate representation of nursing contributions within health care systems. The data generated by this study could also support the development of targeted interventions to improve the visibility and recognition of nursing roles in similar settings internationally.

While this study focuses on NLCs and S&T services in the Italian health care context, the findings may have broader relevance for countries with similar health care structures where nursing roles and documentation practices are evolving.

Differences in health care systems, regulatory frameworks, and the recognition of nursing roles across countries may impact the generalizability of our results. However, the challenges of documenting and recognizing HNAs are likely to resonate globally, suggesting that insights from this study could inform documentation practices and policy reforms internationally. Nonetheless, due to variations in health care policies and the degree of autonomy granted to nurses in different countries, caution should be exercised in generalizing these results. Further research in diverse settings would be beneficial to determine the applicability of these findings to other health care contexts and to explore tailored strategies for improving nursing documentation and visibility worldwide.

### 5.3. Implications for Nursing Education and Professional Development

This study has important implications for nursing education and continuing professional development by highlighting the need to integrate training on documentation practices, finally improving the recognition of HNAs. Enhancing awareness and skills in these areas can prepare nurses to accurately represent their contributions, fostering greater visibility and acknowledgment within health care systems. Furthermore, emphasizing HNAs in educational curricula and professional development programs can equip nurses with the competencies needed to adapt to evolving health care demands, ultimately supporting more effective patient care and organizational efficiency [28].

## 6. Conclusions and Recommendations

This study is expected to provide foundational insights into the scope of HNAs within nurse-led clinics (NLCs) and S&T services in Italy. By systematically documenting and quantifying these often unrecognized activities, the study aims to reveal the substantial role that nursing care plays in patient outcomes, resource utilization, and health care efficiency [29].

These findings will help determine whether there is a need for policy reforms that incorporate standardized nursing terminology and accurate reporting practices to ensure that nursing contributions are visible, measurable, and adequately funded. Establishing a standardized taxonomy specific to NLCs and S&T services would enhance the visibility of nursing practice, allowing for the precise documentation of nursing tasks [3]. Adjusting reimbursement models to reflect the value of nursing-specific activities would align financial compensation with nursing’s impact, creating a more accurate and efficient allocation of health care resources [30]. Furthermore, properly categorizing nursing tasks could reduce waiting times, length of stay and improve patient access, by establishing dedicated nursing service pathways, as already verified in the community nursing-led unit [31], ultimately enhancing system efficiency.

### Study Contribution

This study holds the potential to transform nursing science, health care policy, and public health by enlightening the critical yet often hidden contributions of nursing within Italy’s health care reimbursement system. By identifying, measuring, and documenting nursing work—currently treated as fixed hospitalization costs in Italy [32]—this research will provide robust evidence on the value of nursing care, which is essential for guiding health, economic, and social policy reforms.

This evidence could serve as a foundation for health care reforms that integrate nursing contributions into national financing models, shifting nursing from being a subsumed, fixed cost of hospitalization to a visible, quantifiable, and essential element of health care delivery. By elevating nursing to a more visible and quantifiable level, this research advocates for the development of a standardized nursing taxonomy specific to NLCs and S&T settings, ensuring that nursing practice data are accurately captured and reported [3,33].

The study’s findings are expected to reveal the full scope of nursing work within NLCs and S&T services, much of which is currently misclassified as medical specialist tasks, which inflates waiting lists and delays care. Properly recognizing and attributing these tasks to nursing would support the creation of dedicated waiting lists for nursing services, enhancing service efficiency and allowing for a more accurate allocation of health care resources. Such visibility could reduce pressure on the health care system, demonstrating a direct link between properly recognized nursing activities and improvements in patient access to care.

Beyond health care delivery, this research is positioned to guide strategic planning and resource allocation within health care organizations and at national and governmental levels. The broader impact of this research lies in its potential to inform policy changes that support equitable resource distribution, aligning health care financing, staffing, and the educational preparation of nurses with the actual demands of patient care. Ultimately, this evidence can support policymakers in refining health care financing models, reinforcing nursing’s role in improving patient outcomes, enhancing health care system efficiency, and ensuring a more sustainable health care model for the future.

## Data Availability

No new data were created or analyzed in this study. Data sharing is not applicable to this article.

## Public Involvement Statement

No public involvement in any aspect of this research.

## Guidelines and Standards Statement

This manuscript was drafted following the STROBE Statement.
